# Exploring women’s childbirth experiences and perceptions of delivery care in peri-urban settings in Nairobi, Kenya

**DOI:** 10.1186/s12978-021-01129-4

**Published:** 2021-04-19

**Authors:** Jackline Oluoch-Aridi, Patience. A. Afulani, Danice. B. Guzman, Cindy Makanga, Laura Miller-Graff

**Affiliations:** 1 The Ford Family Program in Human Development Studies and Solidarity, Kellogg Institute for International Studies, Keough School of Global Affairs, University of Notre Dame, Nairobi, Kenya; 2 Department of Epidemiology & Biostatistics and Obstetrics, Gynecology & Reproductive Sciences, University of California, San Francisco (UCSF), Oakland, USA; 3 Department of Psychology, Kroc Institute for International Peace Studies, University of Notre Dame, Notre Dame, USA

**Keywords:** Childbirth, Facility delivery, Experience, Person-centered care, Quality of care, Kenya, Sub-Saharan Africa

## Abstract

**Background:**

Kenya continues to have a high maternal mortality rate that is showing slow progress in improving. Peri-urban settings in Kenya have been reported to exhibit higher rates of maternal death during labor and childbirth as compared to the general Kenyan population. Although research indicates that women in Kenya have increased access to facility-based birth in recent years, a small percentage still give birth outside of the health facility due to access challenges and poor maternal health service quality. Most studies assessing facility-based births have focused on the sociodemographic determinants of birthing location. Few studies have assessed women’s user experiences and perceptions of quality of care during childbirth. Understanding women’s experiences can provide different stakeholders with strategies to structure the provision of maternity care to be person-centered and to contribute to improvements in women’s satisfaction with health services and maternal health outcomes.

**Methods:**

A qualitative study was conducted, whereby 70 women from the peri-urban area of Embakasi in the East side of Nairobi City in Kenya were interviewed. Respondents were aged 18 to 49 years and had delivered in a health facility in the preceding six weeks. We conducted in-depth interviews with women who gave birth at both public and private health facilities. The interviews were recorded, transcribed, and translated for analysis. Braune and Clarke’s guidelines for thematic analysis were used to generate themes from the interview data.

**Results:**

Four main themes emerged from the analysis. Women had positive experiences when care was person-centered—i.e. responsive, dignified, supportive, and with respectful communication. They had negative experiences when they were mistreated, which was manifested as non-responsive care (including poor reception and long wait times), non-dignified care (including verbal and physical abuse lack of privacy and confidentiality), lack of respectful communication, and lack of supportive care (including being denied companions, neglect and abandonment, and poor facility environment).

**Conclusion:**

To sustain the gains in increased access to facility-based births, there is a need to improve person-centered care to ensure women have positive facility-based childbirth experiences.

**Supplementary Information:**

The online version contains supplementary material available at 10.1186/s12978-021-01129-4.

## Background

Maternal mortality has decreased globally. Sub-Saharan Africa, however, still remains the region with the highest contribution to global maternal mortality, with an estimated maternal mortality of 546 per 100,000 deaths. Approximately 66% of all global maternal deaths occur in this region [1]. In 2014, Kenya’s maternal mortality ratio was estimated at 362 per 100,000 live births [2]. Kenya has shown little progress in meeting the MDG targets in 2015 and is off-track to meet the 2030 SDG targets of reducing maternal mortality to approximately 140 deaths per 100,000 [3]. Facility-based childbirth with skilled birth attendance has been identified as an effective strategy for reducing maternal mortality [4, 5]. The proportion of women delivering within a health facility has been increasing globally, including in low and middle-income countries and in sub-Saharan Africa [6, 7]. However, recent evidence in Kenya suggests that these increases in utilization of facility-based delivery have not been accompanied by an improvement in maternal health outcomes [8]. There are also disparities in maternal health outcomes with women of lower socioeconomic status suffering from substantially higher maternal mortality rates [9].

In 2016 research evidence suggests a rise in reports of poor-quality delivery care with only 46% of health facilities in Kenya were reported to have signal functions for emergency obstetric and neonatal care (EMonC) and delivery services [10]. Poorer, unemployed, illiterate, and unmarried women often experience the lowest quality care [11]. Regional variations in quality of care have also been reported, with women in coastal Kenya receiving poorer quality delivery services as compared to city settings. The same study also showed that high-volume health facilities provide better quality of care [12]. Further, several studies in low and middle-income countries (LMICs), including in Kenya, have documented mistreatment and disrespect, and abuse as a challenge for women during childbirth—highlighting gaps in person-centered maternity care [13–16]. Studies examining women’s experiences with quality of delivery care in peri-urban settings have also shown that women valued low-cost unregulated private health facilities because they are responsive to women’s economic and socio-cultural sensitivities for example they invest time when relating with women hence building their confidence [17]. Most studies conducted in peri-urban settings in Kenya have assessed women’s satisfaction with delivery services, [18, 19]. Other qualitative studies have assessed women’s experiences with obstetric emergencies in these settings [20]. Less understood within peri-urban settings is women’s perceptions of person-centered maternity care (PCMC).

Poor PCMC can directly lead to maternal deaths through inadequate identification and management of complications or indirectly lead to maternal deaths through reduced demand for delivery health services [21], 22. In particular, women’s experiences during childbirth are a powerful determinant of the use of maternal health services. It is therefore important to identify ways of promoting high-quality care that is person-centered, particularly in contexts where maternal mortality is high [23]. This study aims to extend the literature by exploring the facility-based childbirth experiences of women living in peri-urban settings in Kenya. Understanding women’s perceptions and experiences of the quality of care can assist both health care workers at facility level and policymakers to enact changes to improve quality of care and subsequently maternal health outcomes. This study was conducted at the beginning of a global pandemic of COVID-19. During the course of the study, the Kenyan health system began to put forward strategies for mitigating the spread of the COVID-19 virus. These findings will therefore also be useful in informing any changes in quality of care due to challenges during the COVID-19 pandemic.

## Methods

### Study setting

This qualitative study was part of a mixed-method study of person-centered maternity care amongst women living in the informal settlements of the Embakasi area in Nairobi City, Kenya. The quantitative study is aimed at measuring PCMC using a validated scale to assess its impact on children’s developmental outcomes. The qualitative study described women’s childbirth experiences to assess their perceptions of PCMC and what contributed to positive and negative experiences, to provide a more holistic understanding of PCMC within the informal settlement context.

Embakasi is the most populous peri-urban area in Nairobi with a population of almost one million people. It is divided into five sub-counties; Embakasi-East, Embakasi-North, Embakasi-West Embakasi-South, and Embakasi Central. The area is characterized by low-income housing, informal settlements with poor access to water and waste disposal. The largest garbage fill for the city of Nairobi is situated in one of the sub-counties, Embakasi-East. The residents who live in this area belong to the lowest wealth quintile in Kenya, and there is widespread poverty and high unemployment.

The health system consists of public maternity hospitals, health centers, and private faith-based health facilities. Faith-based health facilities are owned and run by religious groupings and are classified as private health facilities by the Government. The main public referral health facility for maternity services is a hospital in Embakasi-West.

## Data collection

### Study design, recruitment, and participants

We used a descriptive, qualitative study to explore the experiences of women during childbirth at six different health facilities across the five sub-counties of Embakasi. The data were collected between March and May of 2020 by three research assistants and the first author. The facilities were purposively selected to represent a diverse set of health facilities such as public (both health centers and secondary maternity hospitals) and private health facilities. The health centers are typically operated by midwives and nurses. Medical doctors are contacted on an emergency basis. The maternity hospitals and the main referral health facility are staffed by medical officers and specialist doctors such as obstetrician/gynecologists and pediatricians. The wards in the public health facilities are typically rooms with an average of 8 to 10 beds. The referral health facility has larger maternity wards that can accommodate more women (See Table 1). We recruited women during child welfare clinics. These clinics typically occur a few days each week and provide services such as vaccinations and growth monitoring. The inclusion criteria were women who were aged between 18 and 49 and had delivered their babies within 6 weeks in one of the identified facilities.

**Table 1:** Characteristics of the health facilities where the women in this study delivered

Health Facility	Description							
Health Facility Code	1	2	3	4	5	6	7	8
Facility Level	Health Centre	Hospital	Health Centre	Health Centre	Health Centre	Health Centre	Health Centre	Health Centre
Facility Type	Faith-based	Public	Private	Private	Private	Private	Private	Private
Number of wards	2	4	2	1	2	2	2	2
Staff supporting the wards	Midwife Clinical Officer	Midwife Medical Officer	Midwife Clinical Officer	Midwife	Midwife Clinical Officer	Midwife Clinical Officer	Midwife Clinical Officer	Midwife
Mode of delivery Available	Normal	Cesarean section and Normal	Normal	Normal	Normal	Normal	Normal	Normal

Written informed consent was obtained from all the women after providing information about the study and the potential benefits and risks of their involvement in the study. The women were then asked to share their childbirth experiences. We used a semi-structured in-depth interview guide for the interviews (see Additional file 1: Appendix SI), which were conducted in Kiswahili, a language commonly spoken by women in this setting. The discussions were tape-recorded, transcribed, and translated into English by research assistants and the first author, who are native speakers of Kiswahili. The interviews were also back-translated by one research assistant, a native speaker of Kiswahili, to ensure that the translations were consistent in meaning. A total of 70 interviews were conducted. Of these 30 interviews were conducted in private rooms within the health facilities to safeguard privacy and 40 were conducted via phone because of the restrictions on movement imposed during the COVID-19 pandemic. We obtained ethical review approval from Strathmore University IRB, University of Notre Dame IRB and permission to conduct the research from The National Commission on Science Technology and Innovation (NACOSTI).

### Data analysis

We read all the transcribed data for familiarization. We then entered the data in *Nvivo* software. (QSR International). We followed Braine and Clark’s approach of thematic analysis (2006). We analyzed the data by applying an inductive approach where we allowed themes to emerge from the coded data. We then established categories based on the codes compared these to the interview guide. (See Additional file 2 for the codes) We compared the categories, searched for themes, reviewed themes, and defined the themes identified according to a coding framework, see additional file 3 for coding framework. Lastly, we produced an analysis memo with relevant quotes to support the described themes. When there was disagreement on the themes the two coders JOA and CM compared them and discussed until a resolution was reached. We then compared the themes obtained from the analysis to the three domains of experience of care used in the World Health Organization (WHO) Quality of Care Framework for maternal and newborn health [24] and labeled the themes accordingly.

## Results

The sociodemographic characteristics are presented in Table 2.

**Table 2:** Sociodemographic characteristics of women who gave in-depth interviews N = 70

Participant characteristics	Percentage N (%)
Age (SD)	29 (0.5)
Parity	
Primiparous	16 (23%)
Multiparous	54 (77%)
Marital status	
Single	9 (13%)
Married	61 (87%)
Occupation	
Employed	18 (26%)
Unemployed	52 (74%)
Religion	
Other Christian groups	55 (79%)
Catholic	14 (20%)
Muslim	1 (1%)
Delivery facility type	
Public health facility	28 (40%)
Private health facility	22 (31%)
Faith-based health facility	20 (29%)
Total	70

Four main themes emerged from our analysis. Women had positive experiences when care was person-centered—i.e. responsive, dignified, supportive, and with respectful communication. They had negative experiences when they were mistreated, which was manifested as non-responsive care (including poor reception and long wait times), non-dignified care (including verbal and physical abuse, lack of privacy and confidentiality), lack of respectful communication, and lack of supportive care (including denial of companions, neglect, and abandonment during care, and poor health facility environment).

### Positive experiences

Positive experiences fall under four themes: responsiveness, dignified care, respectful communication and supportive care.

### Responsiveness of the health facility

Responsiveness is the ability of the health facility to meet the population's legitimate expectations regarding their interaction with them**.** Women described positive experiences when they were welcomed at the gate by either the watchmen or the healthcare workers to the health facility. This reception at the first contact highlighted the level of responsiveness of the health facility. This was especially critical when women reported to health facilities at night during active labor.

*“...When I reached there, they welcomed me well, because I reached there at night the received me from the gate and they brought me to the doctors they served me well…”*

*(Respondent #4, Private health facility)*

Responsiveness was also manifested by timeliness of care and healthcare workers’ availability and readiness to provide women with services at their time of need. Women were happy when they had short waiting times, especially during labor. They especially appreciated timeliness in the event of an obstetric emergency.

*“...They don't keep you waiting the moment you get in there, so you don't wait for long.”*

*(Respondent #58, Faith-based health facility)*

*“...when I arrived at the facility, I found the nurse was ready for me. The nurse gave me some injections and instructed me to contact him when I noticed any changes. When I called on him, he came immediately and helped. After delivery, I took a shower and the baby was brought to my bed. I was very happy and impressed with the services…*”

*(Respondent #1, Faith-based health facility)*

Responsiveness ranged from health care worker availability for assistance without complaints, to emergency response through the provision of ambulances for transport to higher levels of care. Women particularly indicated that the health care workers at faith based and private health facilities were available and ready to attend to them. Women noted that when called, they came immediately and were ready to provide assistance. They expressed their satisfaction with and appreciation of the regular attention.

*“… When I called them, they could come, they never insulted me or utter bad things, and they were showing me how I could stay well…”*

*(Respondent #6, Faith-based health facility)*

*“..I can say because their services were good, .they give you food according to the quantity you request and every time the doctors checks his works they keep on coming back to check on you until they make sure you are ok until you leave…”*

*(Respondent #23, Public health facility)*

Extra efforts at providing health education and awareness was also an aspect of responsiveness. Once women had delivered their babies, they appreciated health care workers who assisted them by teaching them how to breastfeed their babies and provide other postpartum services.

*“…the services there even after I finished giving birth, they took the baby well and dressed him well. After that you know also in the ward the way they take care of you, they check on you, they teach you how to breastfeed…”*

*(Respondent #69, Faith-based health facility)*

Lastly, critical to the experience of care, was the availability of ambulances at the health facility and health care workers who were able to access these services and employ them to the benefit of the women.

*“…You see the goodness with that facility is that even if you have a problem, you see you even have ambulances there. So it is very easy to help you and the doctors are very active there is no way you can go through many problems…”*

*(Respondent #69, Faith-based health facility)*

### Dignified care

Dignified care describes care that is kind, respectful and confidential. Women used terms such as “kind”, “friendly, “caring”, “polite” and “concerned” to describe some of the health care workers at some of the health facilities. Kindness was the most common positive quality, with some women describing health care workers who went out of their way to treat them with kindness and provide them with advice on pain management during childbirth.

*“They were kind, teaching on how to behave, what type of food to eat, how to control yourself”.*

*(Respondent #29, Private health facility)*

*“…I liked the way I was served, and the people there are caring, loving, polite and concerned…”*

(*Respondent #29, Private health facility)*

They also described the health workers as “humane” and “professionals” and this made them feel safe in their care. These positive experiences encouraged the women to seek care at the health care facilities as well as encourage their friends and family to seek services at the health facility.

*“…They attend to you in a friendly way and they don’t make you stay there for long. I like it because they treat people nicely, they are humane and generally it is clean. They have good services. I was not delayed at any given moment. There is no harassment.”*

*(Respondent #38, Public health facility)*

*“Yes, I think they are professionals, they know exactly what they are doing... I felt I was safe because they offer good services” I was quite well; it was good since the nurses were always around asking how I feel and how the baby is doing.”*

*(Respondent #29, Private health facility)*

Some women also expressed shock at how the health care workers were kind to them on certain occasions, suggesting that they weren’t expecting such kind treatment, possibly because of a broad culture of unkindness by healthcare workers providing maternity services within this setting.

“…This time they were kind and friendly unlike those other times…’’

*(Respondent #8, Private health facility)*

### Respectful communication

Women interviewed reported that health workers spoke to them in a respectful manner. Some women mentioned choosing the health facility based on how the health workers communicated with them and also from hearing about their friends’ experiences. They reported that they appreciated health care workers who spoke to women respectfully.

*“... They served her well because they were talking to her with respect. That's what made me go there…things like the way doctors talk to people. Talking is how you know health workers' attitudes towards you. In other hospitals doctors talk to people in a bad way…”*

*(Respondent #42, Faith-based health facility)*

They also connected clear communication to assistance in understanding the birth process.

*“…According to me it(communications) means when I get there, I will have someone with me during labor, who will explain to me, what I have to do to feel comfortable, the child is in which stage, you should expect such. Something like that…”*

*(Respondent #21, Private health facility)*

Some women described respectful communication as health care workers who inquired about their emotional state, listened to their concerns about delivery, provided encouragement, advised them and explained to them what was going on.

*“I was quite well; it was good since the nurses were always asking how I was feeling…”*

*(Respondent #21, Private health facility)*

*“…Their services are good they take their time to listen to the patient’s problems...”*

*(Respondent #23, Private health facility)*

Communication that included explanations was especially important to women when told what to expect about birthing, especially when the baby is in distress.

*“…There are Doctors like this one, he told me the baby is tired while inside the womb. This is good, because he told me in advance. There are those who just keep quiet and they know what is going on and after you get a problem is when you ask them later on why they didn’t inform you on what was going on. They then inform you on whether you should have an operation so that they can help you. So, health workers should tell you in advance what is going on and how they can assist you…”*

*(Respondent #65, Public health facility)*

### Supportive care

Supportive care was described by women as allowance of a birth companion during labor and delivery, provision of birthing items, and professional care such as provision of pain medication and good clinical practice. Supportive care also included providing physical assistance such as rubbing their backs to reduce pain during labor and assisting with carrying the baby immediately after birth when they felt weak.

*“... from my experience, they treated me very well. I did not feel pain, only afterwards, however, whenever you told them you were feeling pain, they would give you painkillers whenever I needed anything like a drug they would help. They also treated the baby well. They even showed me how to use some of the medicine I had forgotten to use…”*

*(Respondent #2, Public health facility)*

Some women described good clinical services post-delivery and reported positive experiences because of the meticulous way that the health care workers served them and their babies.

*“…Like the way I had given birth they put for me the baby, they showed me the gender, they put the baby here on the stomach and he slept, they took the baby and washed him well and also the way they stitched me… they did not leave me like that, they washed me. I was bleeding a lot so they injected me and gave me some medicine for pain and advised me on what to do so that would help. They stitched me and told me to treat it with hot salty water for one week. My baby was also cleaned well…”*

*(Respondent #65, Public health facility)*

A few women perceived supportive care as the provision of emotional support such as encouragement as well as information on what stage of labor one was in and other medical information that they needed throughout the process of delivery. They suggested that health care workers should focus on providing moral support and empathy towards women during the delivery process.

*“…When the nurses are close to you and supporting you during delivery, especially encouraging and helping you to push. They give you moral support to the patient. This is also important during delivery, especially the giving of encouragement...”*

*(Respondent #1, Private health facility)*

Some women perceived supportive care as provision of essential birth supplies such as soap and other essential products such as sanitary towels or diapers, and clothes for the baby.

*“… When I got this first baby, I delivered at a public hospital, you were supposed to go with your own things and here I had not carried anything so I felt that they did well. After getting the baby you were given hot water to shower and soap if you did not have…”*

*(Respondent #40, Private health facility)*

Women also felt supported when the facility environment was clean, and they were given sufficient food. In addition, they felt supported when the health care workers secured a support person to escort them home post-delivery or coordinated their referrals to another facility for additional care.

*“…I can say because their services were good, it was clean and they give you food according to the quantity you request and every time the doctors checks his works they keep on coming back to check on you until they make sure you are ok until you leave and even when you are leaving they will not allow you to go alone with the baby they must make sure you have someone to take you up to your house. They can’t also allow you to go if they know you have a certain problem they will tell you to wait so that they can monitor you first and if they feel they are not able to manage your case they will call another hospital and make sure you are treated…”*

*(Respondent #23, Public health facility)*

## Negative experiences

The negative experiences were described under the themes of non-responsive care, non-dignified care, poor communications, and lack of supportive care including poor facility environment.

### Non-responsive care

Non-responsive care manifested itself in various ways including poor reception at the facility, long wait times before being attended to and non-responsiveness of health workers to their needs.

Women described several negative experiences with regard to the level of responsiveness at the health facilities. First, instead of a warm reception at the gate of the health facility, some women reported that they were denied entry into the health facility and asked to go back home. One woman who went into labor early in the pandemic described being asked to wait out in the cold near the gate area. She mentioned that the watchman did not explain to her the reasons for asking her to wait in the cold as elaborated in the quote below;

*“…Yes, we sat down there at the gate. In the tents, that’s where we were seated, because even the watchman was chasing us away. I told him even if it is not an ambulance even a private car could help, he said there is no way I could get a car and it was already curfew time. I asked him what I should do because it was already at night and he did not want to get me an ambulance and he refused to admit me then I asked him If I can get somewhere to sleep till morning so that I can see where I can go, he told me I will sleep in the cold inside there and that I should look for somewhere else to sleep…”*

*(Respondent #60, Public health facility)*

Women described constantly being turned back at the health facility when they believed they were in active labor. The women reported disputed delivery timings when doctors asked them to leave whilst they felt that they were due. The women perceived this as lack of responsiveness.

*“…Yes, I saw the doctor. He then told me that I still wasn’t ready for delivery and told us to go home. But because my water broke, and the pain I was in was a lot, my husband said we shouldn’t go back home; so, we sat down there until around 4 am when I was induced and went up to the maternity room…”*

*(Respondent #60, Public health facility)*

Some women described long waiting times for services such as scheduled cesarean sections. They mentioned that because of the number of other women at the admissions they were kept waiting, but even when the number of women subsided, they were still kept waiting without explanation for when they would receive the services. Some women described waiting for four hours or more. They eventually sought transfers and were only provided with services when they threatened to leave.

*“…They did not attend to me well, because I had to wait for too long before being attended to, and the injections they gave me were late…*

*(Respondent #60, Public health facility)*

*“…They should also improve on admittance; they take a lot of time which is unnecessary. You can wait there for almost 8 hours. Some women would get to the point where they almost deliver and they are yet to be admitted…”*

*(Respondent #21, Private health facility)*

*“… I did not feel supported, because they let me wait for too long and only helped me in the last minutes when I was already angry and looking for a transfer of which they refused to grant me the permission to leave the health facility…”*

*(Respondent #21, Private health facility)*

Some health workers were not only unresponsive, they also explicitly told women not to call them for help and admonished them any time they tried to reach out for assistance.

*“…In that room I was there was another lady who was giving birth and the nurses were on the other side they said you should never call them until you feel like going for a long call, and they are women like you [and] they should understand…”*

*(Respondent #41, Faith-based health facility)*

*“... there were four beds and now when you feel too much pain and call they do not come, when you call them they say you should not call them they know their time when you should call them, you are in pain there you call them, they don’t come … so when you try to get out they tell you to go back time has not reached…”*

*(Respondent #64, Public health facility)*

Some women described health care workers who only worked when their supervisors were around. Women only got a sense of comfort if they could complain to the health workers’ supervisor about the mistreatment.

*“…The supervisor should be around so that we can complain to them whenever we have a problem. Whenever she came around, they would attend to us very well, so she should always be nearby in case we are being mistreated we report them…”*

*(Respondent #60, Public health facility)*

Some women seemed to attribute the lack of responsiveness to the shortage of staff to attend to women in the labor and delivery wards. They recommended that the facility employ more health providers with the desired skills and knowledge to improve delivery services**.**

“The department that serves the clinic part lack adequate personnel. They should be many in number to serve the many women who are always attending clinics. Like in my case, I would be kept waiting from 9.00 am to 1.00 pm. That is draining, otherwise all sectors were okay…”

*(Respondent #33, Private health facility)*

### Non-dignified care

Non–dignified care represents care that is disrespectful and abusive, including verbal and physical abuse, lack of confidentiality, and non-consensual care. Verbal abuse was mainly reported to be done by nurses who are primarily responsible for managing labor and delivery at lower level health facilities. The women described being castigated harshly during delivery while expressing pain.

*“…When you are in pain, they were talking harshly telling you not to shout, throw dirt everywhere, put on cotton wool, not to go to the toilet, and if you feel like going to the toilet don’t call them they did not want anyone to cry loudly of pain…”*

*(Respondent #40, Private health facility)*

Some woman described instances of physical abuse such as beatings by health care workers at larger maternity hospitals.

*“…Another time, another lady next to me, had been stitched, but the thread was undoing itself, the nurse then found when a part of it had come undone, when she was administering drugs to us, she then pulled it out, slapped her thighs and told her that she will remain like that without drugs for the rest of the day.”*

*(Respondent #17, Public health facility)*

### Perceptions of women towards undignified care

We also presented women with different scenarios to test if different forms of undignified care such as verbal abuse and physical abuse by health care workers were acceptable to them. Around 80% of the women found both verbal (71%) and physical abuse (74%) unacceptable under any circumstance.

*“…No, it’s not acceptable, I wouldn’t feel good about it. I would just keep quiet because I need assistance but I wouldn’t like it at all …”*

*(Respondent #9, Public health facility)*

*“…No, it’s unacceptable. The women came there to be helped; it wouldn’t be good if they let there without getting help. It would be a big letdown…”*

*(Respondent #21, Public health facility)*

They also found forms of physical abuse unacceptable, such as pinching and slapping during delivery. They noted that women already undergo a lot of pain and suffering; therefore, they should not be subjected to additional pain.

*“… That is very bad because during delivery you push with a lot of pain and you are suffering while been pinched or slapped, it’s not acceptable…”*

*(Respondent #6, Faith-based health facility)*

However, about one in five women thought that both verbal abuse and physical abuse was acceptable in the case the woman’s actions were about to harm the baby. They perceived the shouting as helpful in berating them for disobedience to health care workers, and as encouraging to them to push the baby out.

*If she shouts with an intention of explaining to me what to do when the baby is coming out it is ok. Maybe you are doing the opposite thing and squeezing the baby to death or you are in a bad position. The shouting is intended to stop you from doing something bad. But shouting to scare someone is bad.*

*(Respondent #65, Public health facility)*

*“…Shouting is acceptable, because sometimes you may see that they are disturbing you but they are helping you, so they must shout at you, so that at least you become careful …”*

*(Respondent #69, Faith-based health facility)*

They also believed that physical abuse was acceptable and appropriate in the event that women were not following the instructions of the health worker and would harm the baby. This was especially during labor or crowning.

*“…A woman can be slapped when she is not following the instructions of the Doctor when she is in labor. If it is me, I get angry but I later I forgive those…”*

*(Respondent #66, Faith-based health facility)*

*“…Yes, she can be slapped, when the head of the baby is out and the women puts her legs together, at that moment you slap the woman to save the life of the baby…”*

*(Respondent #61, Public health facility)*

*“… You see when you are giving birth and the baby’s head is almost coming out and you tighten your legs that is where the Doctor may get angry and slap you or open your legs because you may hurt the baby or yourself. That is when they can slap…*

*(Respondent #23, Public health facility)*

Some women described that during their delivery there was lack of privacy and confidentiality because there were no curtains partitioning the wards. Women, especially those who delivered at secondary maternity health facilities, asked for health facilities to provide curtains for partitioning of beds in the labor rooms. They also asked for separate beds and requested for health facilities to end the practice of sharing of beds.

*“…They should improve on the privacy by providing curtains, they should also provide nets, to keep out the mosquitoes and the sharing of beds should stop” …*

*(Respondent #2, Public health facility)*

### Lack of respectful communication

Women reported experiencing poor communication in their interactions with health care workers. They reported that some health care workers often did not communicate with them well and did not seek to understand them and often castigated them when they were in pain. Other common forms of disrespectful communication included scolding women and blaming them for expressing themselves during labor.

*“...A woman should be talked to nicely and as a doctor you should understand the way she is at that moment… because sometimes a woman can be told to lie on the bed and she refuses, this is because labor pains are never the same.*

*(Respondent #60, Public health facility*)

Women noted that some of the nurses and doctors did not allow them to ask questions. Hence the communication was one way and hierarchical. They felt that they were afraid to ask questions especially when they were unsure of something. They also described receiving rude answers when they eventually got the courage to ask the questions with nurses employing language that was deemed disrespectful.

*“…The language of the nurses should change, the women ask questions because they don’t know, and it’s only fair for them to answer women in a respectful manner…”*

*(Respondent #21, Public health facility)*

Occasionally, the problem was a complete lack of communication. The nurses would sometimes completely ignore women who had received interventions such as cesarean section and needed further instructions on specific do’s and don’ts. One such example was a woman whom nurses ignored in the ward after a cesarean section. The woman was only castigated when she attempted to eat foods that were deemed dangerous to her post-surgery status.

*“…I personally read the nurses attitudes and avoided them, but the other women who were next to me really had it rough. She had experienced her first caesarean section and she did not know what to eat and what not to eat or in which positions they were to siting. When she asked a question, she was answered very rudely by the nurses. When she was found eating something she was told- ‘just eat you will die and be taken home by your folks…”*

*(Respondent #21, Public health facility*)

### Lack of supportive care

Lack of supportive care manifested itself in several ways. These include the denial of a birth companion, neglect and abandonment during labor and delivery and denial of essential birthing items and basic necessities such as bathing water and food. Women reported that despite coming to the health facility with a birth companion, the healthcare workers barred support companions during both labor and delivery. They cited health policy that disallowed birth companions because of lack of sufficient space in the wards and privacy constraints at the health facilities. Women who came with birth companions for support were told to ask them to leave.

*“...I wanted to be there with a friend or my husband but most of the time my husband is at work, so I decided to go with my friend. When she arrived with me, she was told here we do not allow people to stay, and was asked to leave, the health worker promised to call her after the delivery to come take me…”*

*(Respondent #22, Faith-based health facility)*

*“… There is another lady I found there who had been brought by another lady, a friend of hers I presume but she was told to go away. They wanted you to stay alone*

*(Respondent #40, Public health facility).*

We also assessed women’s perceptions of supportive care during labor and delivery. Some women did not have a clear understanding of what supportive care constituted. They perceived support during labor and childbirth as unnecessary. They claimed that they did not need support companions because they did not want their spouses or accompanying friends and family to be with them. They expressed fears about being naked around the support person, which would have caused them undue embarrassment.

*“No (laughter) even if it’s my sister for example she is my younger sister came with me for the delivery and I would not want her to see me during delivery, she can talk about what she saw. Only the doctor should see you, if you deliver at home fine because you have no option.”*

*(Respondent #61, Public health facility)*

They said that they felt that companions from family did not have the skills to assist and the presence of qualified health professionals such as the doctor was sufficient for the delivery care.

*“…While in labor so long as you are okay there is no need so long as the doctor is there the most important thing is the doctor, even if you are with someone from your family and they can’t help in any way because they do not have any skills on how to help. It’s good to be with the doctor is the most important person...” (Respondent #59, Public health facility)*

Neglect and abandonment were reported as another indication of lack of supportive care. Women described experiences where nurses failed to assist them and abandoned them at critical moments such as during the actual delivery. This left them feeling helpless as they said that during the actual ‘pushing’ of the baby is when they needed the most assistance and hence, they had to struggle on their own.

*“…I felt bad because they were shouting at you and you are in the same room… they didn’t want to help you to push the baby you had to struggle alone…”*

*(Respondent #40, Private health facility).*

Women described scenarios in their ward where others were ignored and denied clinical care such as during suturing.

*“… Another time another lady next to me had been stitched, but the thread was undoing itself, the nurse then found when a part of it had come undone when she was administering drugs to us, she then pulled it out and told her that she will remain like that without drugs for the rest of the day. Mind you the lady had come the previous day, so she just stayed the whole day without drugs. Whenever we tried to intervene, she asked us if we worked there. That did not go well with anyone of us…”*

*(Respondent #21, Public health facility)*

Women also viewed denial of essential birthing items as lack of supportive care. Women reported asking the health facilities to provide them with basic provisions such as sanitary towels, buckets and hot water for bathing. Some claimed that their requests for sufficient food were denied. They explained that they felt unsupported when these basic items were denied.

*“..After delivery, I was taken to the ward and they told me that food was over and I had to wait until night then they gave me ugali (maize meal) and kales…No I didn’t expect this to happen to me because I believe that there is no hospital you can be told there is no food, because they know people must be fed”*

*(Respondent #48, Private health facility)*

Poor environment such as unhygienic facilities, dirty bathrooms and persistent water shortages were viewed as unsupportive.

*"... When there was water shortage, the toilets would get dirty, making it hard to use them…”*

*(Respondent #2, Public health facility)*

*"... You see after giving birth you feel very cold you even shake. I don’t know where the coldness come from, you are told to go and shower with cold water and where you go to shower there is blood from others who have given birth, it is dirty, the water is cold and you are also shaking… "*

*(Respondent #64, Public health facility)*

Facility cultures that were permissive to practices such as bribery and informal payments to health workers were also reported as contributing to unsupportive and negative experience at the health facility. Some women described bribing as a way to receive better treatment at the larger secondary hospitals.

*"... They attend to you well if you have bribed them. They don’t spend more than 3 minutes away before coming to find out how you are doing. When I got to the labor ward there were women who were being treated better and we were wondering why, until later we came to find out that they had given the health workers money…*

*(Respondent #60, Public health facility)*

Some women reported that they believed that the free maternity under the ‘*Linda Mama’ (protect mothers)* vouchers provided for their entire maternity experience to be free of charge. This voucher was established by the Government in 2013 to provide free delivery services for women in the country at public health facilities [36]. However, they reported being asked to pay for certain services such as the provision of medication. They recommended that health facilities be transparent about the delivery charges.

*"... What did not please me there was that the health facility usually say that giving birth is free, but sometimes when you go there expectant for them to give you some medication they ask you to go bring money first and may be you don’t have, so they will not give you. Again, I hear when you go you are required to u give like KSH 300, (USD 3) and I didn’t know what that 300 Ksh was for because we were told giving birth is free. Now that is what I hated …"*

*(Respondent #12, Faith-based health facility)*

## Discussion

We conducted in-depth interviews with 70 women in a peri-urban part of Nairobi to explore their perceptions and childbirth experiences. Women had positive experiences when care was person-centered—i.e. responsive, dignified, supportive, and with respectful communication. They had negative experiences when they were mistreated, which was manifested as non-responsive care (including poor reception and long wait times), non-dignified care (including verbal and physical abuse lack of privacy and confidentiality), lack of supportive care (including being denied companions, neglect and abandonment, and poor facility environment) and lack of respectful communication. Giving bribes appeared to influence the type of care women received.

Responsiveness has been identified as a core element of a health system that has user experience in mind and constitutes a positive experience in other studies conducted in Kenya [19]. At the maternity level, women in advanced labor who presented themselves at the health facility were sent away from the health facility without any form of clinical assessment. This was sometimes enforced by support staff, including guards at the gate. These findings concur with our findings that emphasize the role of all staff, including auxiliary staff such as cleaners and watchmen, as critical to women’s experiences of PCMC [25]. Other studies have also identified responsiveness as a key component of a health system and related to how health care workers respond to non-clinical needs of women [26].

Undignified care during maternity has been identified as a growing problem particularly in low and middle-income countries [21]. Forms of undignified care such as verbal and physical abuse, neglect and abandonment have been identified in Kenya, as well as other settings in sub-Saharan Africa [13, 16, 27]. Within this setting, undignified care was overwhelmingly identified as happening at larger secondary public maternities, which is consistent with findings from other studies [11, 28]. Some forms of undignified care such as physical abuse have been attributed to health system conditions that include overcrowding and understaffing [16, 29]. They have also been attributed to health worker attitudes that drive them to physical abuse as a means of gaining control and compliance over women [30]. Lack of privacy in the wards was also identified as a type of undignified care; women recommended partitions to the beds and called for the abolishment of sharing of beds. This has been suggested in several studies so that women can access care that is perceived as dignified [25].

Most respondents in this setting reported that they were accompanied to the health facility by friends and family; however, birth companions, who are supposed to provide continuous support during labor and delivery, were not allowed to stay with the women. Health workers cited privacy concerns within the labor ward because of the small spaces and overcrowding in the wards. Other studies conducted in Kenya have reflected similar findings where women were not allowed support companions; however, this study deviates from similar studies in rural areas, where health workers cited lack of trust for the accompanying friends and relatives [31]. Instead, in our setting most of the women indicated that they were not interested in having a support companion present during delivery. This finding is congruent with other studies conducted in Kenya where women did not want birth companions [31]. They provided reasons such as embarrassment and cited that health workers were sufficient to handle their health care needs. Women had varying perceptions around supportive care; only a handful of women mentioned emotional support as a form of support. This was similar to the aforementioned study in Western Kenya where companions were perceived as providing assistance with other non-clinical tasks rather than emotional support to women [31]. We suggest that for support during labor and childbirth to be embraced by the health system, women need further education during antenatal care (ANC) on the role of emotional support in securing good outcomes during delivery, but that providers should be attuned to how this support can be provided within the bounds of women’s preferences related to privacy and comfort. It also requires a health system that views support during labor and delivery as a quality improvement similar to improvements in physical space and privacy provision. Continuous support during labor has been proven in other contexts to promote safe deliveries including increased spontaneous vaginal birth, shorter duration of labor, and decreased caesarean birth, instrumental vaginal birth, use of any analgesia, use of regional analgesia, low five‐minute Apgar score and negative feelings about childbirth experiences [32].

Neglect and abandonment was a common form of unsupportive care identified. Women described being left alone during labor and birth, as well as provider failure to monitor them and intervene in life-threatening situations. These practices were seen in other studies in Kenya and other LMICs [14, 33]. This practice runs counter to WHO standards for high quality services for women and children during child birth. These standards require health facilities that need to have minimum standards of care that promote evidence based practices that can situate labor timing and provide continuous support during labor and delivery [34].

Unhygienic facility environments especially at bathrooms and wards were identified in this setting. This was particularly common in public health facilities. Such conditions have been identified as a deterrent to women’s satisfaction with the facility-based delivery in peri-urban settings in Kenya [19]. A facility culture of bribery and informal payments was identified at public hospitals with large volumes. Other studies have identified corruption in larger health facilities in Kenya [33]. Women provided recommendations for health facilities to be transparent especially during the era of free maternity services. They requested that facility managers create channels for redress when women are over charged or are asked for a bribe. They also demanded clarity around additional auxiliary out-of-pocket charges.

A few women in this study identified gaps regarding respectful communication in interactions with health care providers. Women mentioned that health care workers did not listen to them or allow them to ask questions about their care. Women need to be given information about their birth and allowed to assess progress. Listening to individual concerns will assist health care workers to make the correct decisions for their care during labor and delivery and should be prioritized during quality improvements. The WHO standards for care require that women receive communication that is effective and that responds to their needs and preferences [34].

Interventions aimed at improving the quality of services during facility-based care should focus on promoting positive experiences and mitigating negative experiences. For undignified care, recommendations have been made on promoting accountability of health care workers [35, 36]. Forms of accountability include administrative and bureaucratic approaches, patient-oriented approaches such as a consumer forum, and approaches which enforce standards of care such as posting a bill of rights on the walls and suggestion boxes [33]. Anonymous call systems can allow women to report breaches in adherence to high quality standards of care. Retraining of healthcare workers using transformational strategies that examine value systems has also been recommended [13]. In order to promote supportive care, health systems need to change their policies and promote the acceptance of birth companions as they are known to improve birth experience [32]. Other forms of support such as provision of birthing items and a facility environment that is conducive would assist with improving women’s environment. Re-training of health care workers in respectful communication and empathy have also been suggested in trainings that focus on transformative values [13].

### Limitations of the study

The study sites were all in a peri-urban setting of a city and hence the findings are specific to women’s experiences within a peri-urban setting. Results might not reflect the experiences of rural and other women and hence may not be generalizable to other settings in Kenya. Other limitations include potential under reporting of negative experiences due to social desirability bias. This limitation especially pertains to interviews conducted at the health facility Also, women were unable to assess technical quality of care and hence their description of their experiences was solely based on their perceptions of interactions with health care providers. Further research on the perspective of the health care workers could improve understanding of how institutional structures and processes can be reorganized to provide better person-centered care.

## Conclusion

Our study suggests that women experience both positive and negative experiences at health facilities within this peri-urban setting in Kenya. Several gaps in PCMC exist including non-responsive care, undignified care, disrespectful communication and unsupportive care. Positive experiences include dignified and respectful maternity services; these can be used to train health care workers on what women want to have a person-centered experience. Interventions on the health systems can focus on improving health facility cultures. Interventions can range from tackling low-hanging fruit through the provision of essential birthing items, ensuring the cleanliness of health facilities, to addressing more complex items such as addressing health care worker attitudes and retraining health care workers for empathy. Further upstream management issues such as staffing at health facilities, and feedback mechanisms for reporting neglect should be enforced at health facilities. These improvements will lead to higher quality care that can in turn lead to positive maternal outcomes. PCMC quality improvements have been demanded by women, now they should be provided by the health system to improve women’s childbirth experiences.

## Supplementary Information

**Additional file 1**. Indepth Interview Guide.

**Additional file 2**. Open Codes.

**Additional file 3**. Coding Framework.

## Data Availability

The de-identified data are available upon reasonable request to the corresponding author.

## Abbreviations

MDG: Millennium development goals
SDG: Sustainable development goals
PCMC: Person centered maternity care
COVID-19: Coronavirus disease
ANC: Antenatal care
LMICS: Low and Middle-Income Countries
WHO: World Health Organization
