# Supplementary material for: Exploring women’s childbirth experiences and perceptions of delivery care in peri-urban settings in Nairobi, Kenya
Source: Reprod Health. 2021 Apr 19;18:83. doi: 10.1186/s12978-021-01129-4 (PMC8054117; doi:10.1186/s12978-021-01129-4)
Supplement: Supplementary file 1 — Additional file 1. Indepth Interview Guide. [file 12978_2021_1129_MOESM1_ESM.docx]

**COVER SHEET**

**In--depth interview guide for women of reproductive age who delivered at a facility in the past 12 months**

**Step 1:** Introduce yourself to the participant. Describe the purpose of the interview and how information will be used. Obtain oral/written consent.

**Step 2:**  Ask the participant to identify herself. Interviewer: fill out the information below prior to beginning the interview.

**Step 3:**  Conduct the interview. Please remember to audio record the interview.

**Participant information**

**Participant age (write in):**

**Location (urban/rural):**

**Religion (write in):**

**Total number of living children (write in):**

**Total number of deliveries (write in):**

**Number of deliveries in a facility (write in):**

**Number of deliveries outside the health facility (write in):**

**Name of health facility delivered at (write in):**

**Cadre and position (write in):**

**Marital status (Single, married/cohabitating, divorced, widowed):**

**Interview date:**

**Start time:**

**End time:**

**Interviewer:**

**Interview discussion guide**

**A. Childbirth narrative**

1. Tell me about your birth story? When did you find out you were expecting a baby?
2. Please tell me about your most recent childbirth. I would like to know the most memorable parts – both good and bad. Were you excited to learn that you were pregnant? What were your concerns, worries, fears and joys?
3. Where did you attend your antenatal care services from? How many times did you attend clinic before your delivery?
4. How did you like the facility? What was the quality of care?
5. Who was involved in making this decision about where you would go to give birth?
   - 1. How were they involved?

1. Were you planning to deliver in a health facility? Why or why not?
   - 1. What about the health facility where you delivered? Why did you go there? [Probe: were you referred to/from another health facility or elsewhere?]
2. Did you deliver vaginally or by caesarean section?
   - 1. Did you want this mode of childbirth, or did you prefer something else? Why or why not?

1. Was your baby healthy when it was born? Or were there complications? Please describe.
2. Approximately how long were you in labor in the hospital before you delivered?
3. Approximately how long did you stay in the hospital after you delivered?
4. Did you have any challenges meeting the expenses related to the childbirth process?
5. Did you feel like you had control over decisions around the childbirth? For example, the position that you delivered in? [Probe: lying on your back, kneeling, squatting or other?]
   - 1. Did the midwife offer for you to deliver in a different position? Please explain.
6. Now I would like to talk to you about the hospital that you delivered in. From the time you arrived until you had to start pushing, what do you remember about your surroundings? (*Interviewer: use the probes below)*
   1. Who was there?
   2. Where were you?
   3. What was the room like?
   4. What did you do while you were in this room (probe: move/walk around, take fluids)?
   5. Who else was there?
7. While you were in labor [during contractions but before pushing], was someone with you besides a health worker? For example, a family member, friend or husband?
   - 1. Probe: what was this person’s role during this time?
8. How did you feel while you were there?
9. Would you deliver in the same hospital again? Why or why not?
10. Would you recommend that a friend deliver in this hospital? Why or why not?
11. Overall, how did you feel about your childbirth in that facility?
12. From the time that you started pushing until the baby came out, what do you remember about your surroundings? (*Interviewer: use the probes below)*
    1. Who was there?
    2. Where were you?
    3. What was the room like?
    4. What did you do while you were in this room (probe: move/walk around, take fluids)
    5. Who else was there?
13. While you were delivering your baby [while you were pushing until when the baby came out], was someone with you besides a health worker? For example, a family member, friend or husband?
    - 1. Probe: what was this person’s role during this time?
14. If there was not someone with you during labor or childbirth, would you have wanted someone to be there with you?
    - 1. Probe: what would this person’s role be?
15. How did you feel while you were there?

**B. Perceptions and experiences of care provided at the most recent facility-based childbirth, focusing on treatment by health workers and the facility environment.**

1. Now I would like to talk to you about your perceptions and experiences of care during childbirth. In your opinion, how were you treated by the health workers during your most recent labor and childbirth? Please explain.
2. How did this treatment that you have described make you feel?
3. Did the type of care that you received meet your expectations? Please explain.
4. Could you describe for me what supportive care during childbirth means to you?
5. Did you feel supported by the staff during your childbirth? Please explain.
   - 1. *If respondent says no, probe:* what could be done to improve this in the future?
     2. *If respondent says yes, probe:* what was the most memorable part of the way that you were cared for?
6. In your opinion, what would you need from your MIDWIFE, NURSE OR DOCTOR in a health facility in order to feel supported during childbirth?

**C. Elements and experiences of mistreatment of women during childbirth**

1. Did you experience anything during your childbirth that made you feel unhappy or uncomfortable?
   1. Could you explain the situation?
   2. Who was involved in the situation?
   3. How were you [friend/family] mistreated?
   4. When did it happen? [Probe: time of day, during labor, during childbirth or postpartum].
   5. How often did it happen? [Probe: just once or more often].
   6. Why do you think this happened to you?
   7. How did this make you feel?
   8. In your opinion, how common is the situation that you described? [Probe: do situations like this happen often?]

*Interviewer: After the woman explains the scenario, ask her if there were any other times or ways that she was mistreated. If she describes another scenario, follow-up with questions 5a-5g.*

**D. Perceived factors that influence how women are treated during childbirth**

1. You mentioned these types of mistreatment: *(Interviewer: restate what type of mistreatment the woman experienced. For example, you mentioned that you were hit or yelled at by the midwife].* In your opinion, what factors influenced how you were mistreated? Please explain.
   1. Probe: Related to supplies (availability of medication, equipment)
   2. Probe: Related to health workers (number of staff, attitude towards patients)
   3. Probe: Related to patient load (number of patients, overcrowding)
2. In your opinion, what could be done so that women are treated better during labor and childbirth?

**E. Acceptability of how women are treated during childbirth**

1. Now I would like to ask your opinion on how you feel about the way that women are treated during childbirth. If a woman was pinched or slapped by a health worker during her childbirth, would this be acceptable?
   1. When would it be acceptable?
   2. How would you feel if this happened to you?

1. If a woman was yelled or shouted at by a health worker during her childbirth, would this be acceptable?
   1. When would it be acceptable?
   2. How would you feel if this happened to you?
2. If a health worker was mean and refused to help a woman during her delivery, would this be acceptable?
   1. When would it be acceptable?
   2. How would you feel if this happened to you?
3. If a health worker physically held a woman down during her childbirth, would this be acceptable?
   1. When would it be acceptable?
   2. How would you feel if this happened to you?

**G. Wrapping up**

1. Is there anything else that you would like to tell me about your childbirth?
2. Thank the participant for their time. Remind them that the information they shared will be kept confidential.
