# Supplementary material for: Exploring women’s childbirth experiences and perceptions of delivery care in peri-urban settings in Nairobi, Kenya
Source: Reprod Health. 2021 Apr 19;18:83. doi: 10.1186/s12978-021-01129-4 (PMC8054117; doi:10.1186/s12978-021-01129-4)
Supplement: Supplementary file 2 — Additional file 2. Open Codes. [file 12978_2021_1129_MOESM2_ESM.docx]

**Appendix II**

**Open Codes**

| **Text** | **Open code** | **Sub theme** | **Theme** | **Experience category** |
| --- | --- | --- | --- | --- |
| “The hospital is very clean. I was very impressed. The cleaners were always working to maintain cleanliness” | Hospital is very clean; cleaners always working to maintain cleanliness | Clean facility | Facility culture/environment | Positive experience |
| “I was given some injection and he told me to walk around. He was just nearby and asked me to call him whenever I noticed any changes” | Given some injection; doctor asked to be called in case of changes | Helpful and attentive health care workers | Responsive health workers | Positive experience |
| No, I would not. The way they people share beds is not good, they do not use curtains for privacy, and mosquitoes bite the children.” | People share beds is not good, they do not use curtains for privacy, and mosquitoes bite | Lack of privacy and confidentiality | Facility culture/environment | Negative experience |
| “Yes, I can. Because there is good service delivery at the hospital. It is clean. And there is good food. During my antenatal clinics, the doctors encouraged me to be strong and positive. They helped my overcome any fears that I had.” | Good service delivery; doctors encouraged me to be strong and positive ;helped me overcome any fears | Encouragement from doctors | Supportive care | Positive experience |
| “No, I was comfortable with the nurse. I did not want someone else apart from the nurse. They would not have done much than the nurses would have done.” | Did not want someone else ;would not have done much than the nurses would have done | Perceived need for supportive care | Supportive care | Negative Experience |
| “When I arrived at the facility, I found the nurse was ready for me. The nurse gave me some injection and instructed me to contact him when I noticed any changes. When I called on him, he came immediately and helped. After delivery, I took a shower and the baby was brought to my bed. I was very happy and impressed with the services.” | , I found the nurse was ready for me. | Readiness to provide services | Responsive health workers | Positive experience |
| “When the nurses are close to you and supporting you during delivery, especially encouraging and helping you to push. Giving moral support to the patient is also important during delivery, especially giving encouragements. Additionally, I was given additional fluids.” | When nurses are close to you and supporting you during delivery ;especially encouraging and helping you push ;I was given additional fluids; giving moral support is important during delivery | Encouragement from nurses to push; moral support during delivery | Supportive care | Positive experience |
| “No, I would not. The way they people share beds is not good, they do not use curtains for privacy, and mosquitoes bite the children.” | the way people share beds is not good; they do not use curtains for privacy; mosquitoes bite the children | Lack of privacy and confidentiality  Inadequate basic amenities | Facility culture/ environment | Negative experience |
| “Yes. I would have loved them to hold the baby for me.” | Hold the baby for me | Role of support person | Supportive care | Positive experience |
| “The nurses treated us well, because whenever you said you had a problem they were there to help. If you were feeling pain, they’d give you painkillers, and if the pain was too much, they would give you injections of stronger painkillers.” | The nurses treated us well; they were there to help; they'd give you painkillers ;they would give you injections of stronger painkillers | Given painkillers and injections | Supportive care | Positive experience |
| “They also treated the baby well. They even showed me how to use some of the medicine I had forgotten to use.” | Treated baby well; showed me how to use some of the medicine I had forgotten to use | Shown how to use medicine | Supportive care | Positive experience |
| “They should improve on the privacy by providing curtains, they should also provide nets, to keep out the mosquitoes and the sharing of beds should stop.” | Improve on the privacy; providing curtains; should provide nets; sharing of beds should stop | Lack of privacy and confidentiality  Inadequate basic amenities | Facility culture/ environment | Negative experience |
| “The way they took care of the baby and their attention to the medicine we needed. Also, they provide hot water for drinking which was required after undergoing CS.” | Took care of the baby; attention to medicine needed; provide hot water for drinking which was required after undergoing CS | Given the necessary attention after undergoing CS | Health care workers providing dignified care | Positive experience |
| “When there was water shortage, the toilets would get dirty, making it hard to use them.” | there was water shortage ;toilets would get dirty; making it hard to use them | Poor hygiene conditions of facility | Facility culture/ environment | Negative experience |
| “They should attend to the women who are in labor, instead of ignoring them scream until the baby comes.” | should attend to the women in labor; instead of ignoring them scream until baby comes | Neglect during delivery | Mistreatment during facility based delivery | Negative experience |
| “Cleanliness, the bed and food was brought for me and I showered with hot water then I was given drinking water.” | Cleanliness; food brought to me; showered with hot water; given drinking water | Given what is required after delivery | Health care workers providing dignified care | Positive experience |
| “*Laughs* … No, that delivery pain cannot allow a family member to be near there.” | Delivery pain cannot allow a family member to be near there | Did not want support person during delivery | Supportive care | Negative experience |
| “The doctors are also good they do not quarrel when you ask they anything they explain to you well.” | Doctors do not quarrel and explain things well | Proper communication and instructions from health workers | Respectful communication | Positive experience |
| “Yes they supported me you see for the baby when you come out of theatre you are not able to cloth that is the first thing I found that they had clothed him very well and I was also clothed well so there is way they assisted me. Like the first one it is me who clothed him with all the pain I had.” | They clothed him very well; and i was also clothed well; they assisted me | Baby and woman gien support | Supportive care | Positive experience |
| “You see the goodness with there even if you have a problem, you see you even have ambulances there so it is very easy to help you and the doctors are very active there is no way you can go through many problems.” | If you have a problem you even have ambulances; It is very easy to get help; The doctors are very active | Adequate facility equipment  Active doctors | Facility culture/ environment  Responsive healthcare providers | Positive experience |
| “Cleanliness there is water there they give you food, at least I was pleased with everything.” | Cleanliness; there is water; they give you food | Clean facility and availability of food | Facility culture/ environment | Positive experience |
| “The services there even after I finished giving birth they took the baby well and dressed him well after that you know also in the ward the way they take care of you they check on you they teach you how to breastfeed everything how you should do they also give you pain killers.” | They took the baby well and dressed him; they check up on you in the wards; they teach you how to breastfeed; they give you painkillers | Baby and mother taken care of after delivery | Supportive care | Positive experience |
| “I was really treated very well and with care, the doctor even gave me some medicine to help boost my blood.” | Treated very well and with care; Given medicine to help boost blood | Cared for and given medication | Health workers providing dignified care | Positive experience |
| “I didn’t see any problem but what made me feel bad was the smell in the labor room.” | What made me feel bad was the smell in the labor room | Foul smell in labor room | Facility culture/ environment | Negative experience |
| “Yes, that is my hospital in Ruben nurses and doctors are welcoming, loving and talk to people with respect.” | Nurses and doctors are welcoming; loving; talk to people with respect | Nurses and doctors speak to people with respect | Respectful communication | Positive experience |
| “I was comfortable, the place is clean doctors tell you what you are supposed to do they are with you all the time while you are in labor pains you’re given a trolley to push.” | I was comfortable; the place is clean; doctors tell you what you are supposed to do; they are with you all the time when you have labor pains; given a trolly to push | Clean facility  Doctors give instructions and are present throughout | Facility culture/ environment  Healthcare workers providing dignified care | Positive experience |
| “The beds were less that is what affected us more materials I did not see any wrong there that is only what I saw because people giving birth are many you may get one bed they even share three people including children you see and those beds you know their size.” | The beds were less; people giving birth are many; you get one bed they may even share three people | Sharing of beds, congested facility | Facility culture/ environment | Negative experience |
| “It is also a government hospital it has enough equipment’s unless before you get referred to another hospital there they have tried a lot mostly it helps many people it has……..it has enough doctors and nurses are there also enough.” | Government hospital; has enough equipment; helps many people; has enough doctors and nurses | Availability of equipment and enough staff | Facility culture/ environment | Positive experience |
| “They are not very harsh, you know there are some doctors or nurses when you are in pain and you know the pain comes with very many things you even get crazy you see but those were talking nicely to people you see they were not talking harshly that do this or do that or even chase you away or even in the room when you make noise they were coming and talking to you in a good way on what you should do you know there are others who scream or talk in their own ways so they were bringing some silence they tell you to persevere then they will help you well.” | Are not very harsh; those were talking nicely to people; they were not talking harshly that do this or do that or even chase you away; they were coming and talking to you in a good way; they tell you to persevere then they will help you well | Understanding health workers; concerned | Respectful communication | Positive experience |
| “Like the way I had given birth they put for me the baby they showed me the gender, they put the baby here on the stomach and he slept, they took the baby and washed him well and also the way I ….they stitched me they did not leave me like that they washed me I was bleeding a lot they injected me and gave me some medicine for pain and advised me what to do so that that wound to heal they stitched me and told me to treat it with hot salty water for one week so if they had not told me that way I could have left it and may be it could have rotten. My baby was also cleaned well I didn’t get any dirt in him they told me to breastfeed the baby…” | They put for me the baby and showed me the gender; they took the baby and washed him well; they stitched me; they did not leave me; i was bleeding a lot and they injected me; gave me some medicine for pain; told me to breastfeed my baby | Took care of the baby and mother | Supportive care | Positive experience |
| “You see after giving birth you feel very cold you even shake I don’t know where the coldness come from, you are told to go and shower with cold water and where you go to shower there is blood from others who have given birth, it is dirty, the water is cold and you are also shaking.” | After giving birth you feel very cold; you are told to go shower with cold water; where you do to shower there is blood from others who have given birth; it is dirty | Given cold water to shower; dirty facility | Facility culture/ environment | Negative experience |
| “The room had a delivery bed, at the corner they had their tools for delivering the babies, there was also a place to put the baby and bathroom” | Had a delivery bed; had their tools for delivering the babies; place to put the baby; bathroom | Well equipped facility | Facility culture/ environment | Positive experience |
| “Yes, definitely. Because the doctors and nurses there treat mothers with dignity, they are gentle and treat you with respect.” | Doctors and nurses treat mothers with dignity; they are gentle; treat you with respect | Respect, gentle and dignified care | Health workers providing dignified care | Positive experience |
| “I was satisfied and grateful. The doctors and nurses treated me with such kindness. The doctors and nurses were more interested in making sure that the baby was born safely without any problems. The kitchen staff also made sure that we were fed. The ward was clean and there was always a cleaner around cleaning**.”** | Doctors and nurses treated me with such kindness; doctors and nurses interested in making sure the baby was born safely; kitchen staff made sure we ate; the ward was clean; there was always a cleaner | Treated with kindness;  Clean facility | Health workers providing dignified care  Facility culture/ environment | Positive experience |
| “I would have wanted my husband to be with me; to help me and support me whenever I needed help. For example, if I wanted an article of clothing from my bag to dress the baby or to help look after the baby when it was necessary to visit the rest rooms. Most importantly however, to give me the emotional support that I needed.” | Would have wanted my husband to be with me; to help and support me whenever i needed help; if i wanted an article of clothing from my bag to dress the baby; to help look after the baby when it was necessary to visit the rest rooms; give me emotional support that i needed | Did not have a family support person | Supportive care | Negative experience |
| “Yes, I was supported by the nurse. As the baby was coming out, the nurse told me how to push so that the baby can come out. She then cleaned and dressed the baby after delivery. “ | I was supported by the nurse; the nurse told me how to push so that the baby can come out; she cleaned and dressed the baby after delivery | Baby and mother supported during and after delivery | Supportive care | Positive experience |
| “I was sleeping on the bed alone, when I pushed I heard the nurse saying this woman will tear the nurse was very harsh when I tried to touch her she told me don’t touch me you have corona.” | When i pushed i heard the nurse saying this woman will tear; the nurse was very harsh; when i tried to touch her she told me i have corona | Verbal abuse Harsh nurse; rude | Mistreatment during delivery | Negative experience |
| “I was not at peace I was wondering what will happen next am alone no relative you don’t know whom you can tell what has happened” | I was not at peace; no relative; you don’t know whom you can tell what has happened | No support companion | Supportive care | Negative experience |
| “No doctors don’t take care or even ask you how you are feeling they just pass you they tell you don’t touch me, cover your mouth with a mask so you fail to understand how things happen” | Doctors don’t take care or even ask you how you are feeling; they just pass you; they tell you don’t touch me | Neglect during delivery | Mistreatment during delivery | Positive experience |
| “in that room when I entered there was a woman who was lying in the bed l was told to lie on next me was a woman haaa there was a smell I don’t know if it was her stool, that hospital is dirty there was a lady with a bucket of water when dirt drops down she wipes.” | There was a woman who was lying in the bed; i was told to lie next; there was a smell; i don’t know if it was her stool; that hospital is dirty | Dirty facility | Facility culture/ environment | Negative experience |
| “He was very happy because he saw the place was good, clean..” | Place was good, clean | Clean facility | Facility culture/ environment | Positive experience |
| “Yes , I stayed the whole day there was a problem of beds. It was said people are many and those who delivered in the morning to leave, yes.” | There was a problem of beds; those who delivered in the morning had to leave | Inadequate basic amenities | Facility culture/ environment | Negative experience |
| “Aaah!......laughs…..they….they gave me blood boosting drugs, they told me how I should eat vegetables, how I will eat four times a day, they told me many things.” | Gave me blood boosting drugs;told me how i should eat vegetables;told me many things | Dignified care | Supportive care | Positive experience |
| “I saw they were…… I mean when I called them they could come, they never insulted me or utter bad things , they were showing me how I could stay well.” | When I called them they would come; they never insulted me or utter bad things | Responsiveness | Responsive health care workers | Positive experience |
| “I did not like the way those women were waiting to be admitted yet they were on labour and inside when a nurse Asked a woman to clean up her mess when she was on pain You know it was not her intention to help herself there.” | Did not like the way those women were waiting to be admitted; a heavy woman is waiting for a long time to be admitted  nurse asked woman to clean up her mess when she was in pain ; | Long waiting times    Neglect during delivery | Facility culture/ environment  Mistreatment during delivery | Negative experience |
| “I remember them calling for more help because the baby came out feet first, they came in quite a number of them. They came very fast.” | Calling for help because the baby came feet first; | Fast response | Responsive health care workers | Positive experience |
| “Maybe they would help in soothing the back pain since I had to request the nurse to do that for me. If any of them is present in the room, it would make it easier.” | Would help by soothing the back pain; it would make it easier | Perceived role of supportive care | Supportive care | Positive experience |
| “Yes, you are crowded others do not have masks and you can touch any place, you see. Sometimes running water gets finished so you have to wash your hands there in the toilet and it is dirty.” | You are crowded; others do not have masks; running water gets finished; the toilet is dirty | Crowded facility; no water; dirty toilets | Facility culture/ environment | Negative experience |
| “They did not attend to well, because I had to wait for too long before being attended to, and the injections they gave me were late.” | Did not attend well; i had to wait for too long before being attended to; injections they gave me were late | Long waiting time | Facility culture/ environment | Negative experience |
| “It was dirty, they even left corpses of the dead babies there. They took too long before coming to even clean the bed where I was.” | It was dirty; they even left corpses of the dead babies there; took too long before coming to clean the bed where i was | Dirty facility | Facility culture/ environment | Negative experience |
| “I felt bad because nobody was attending to me.” | I felt bad; nobody was attending me | Neglect | Mistreatment during delivery | Negative experience |
| “No. Maybe if I go there, I bribe them in order for them to attend to me well. If you give them some cash, they treat you well, make sure you get a bed and stay with you through out.” | If you give them some cash they treat you well; make sure you get a bed; and stay with you throughout | bribery in health facilities | Facility culture/ environment | Negative experience |
| “I was not supported. I sat out in the cold while in labor and got myself where to lay. When the baby was coming out is when they tried to help. I was not helped I helped myself.” | I was not supported; i sat out in the cold; got myself where to lay; i was not help, i helped myself | No support | Supportive care | Negative experience |
| “I was abandoned while feeling pain for a long time. We were also congested; we were overcrowded yet there is covid 19. They should also say if they want money in order to treat us better.” | I was abandoned while feeling pain for a long time; we were congested; we were overcrowded | Neglect during delivery  Overcrowding | Mistreatment during delivery  Facility culture/ environment | Negative experience |
| “They did yell at me. When I said I was feeling pain and needed a pain killer, the attendant asked if my husband had paid for the injection” | They did yell at me; i was feeling pain and needed a pain killer; asked if my husband had paid for the injection | Verbal abuse | Mistreatment during delivery | Negative experience |
| “They were ok serving customers but what surprised me they prescribe medicine for you to buy from outside instead of them giving you there. There are some medicine that they do not have , then I was seeing the medicine you can’t buy because they were saying bring money we give you the medicine.” | What surprised me they prescribe medicine for you to buy from outside instead of them giving you there; there are some medicine that they do not have; I was seeing the medicine you can’t buy because they were saying bring money we give you the medicine | Inadequate medical supplies | Facility culture/ environment | Negative experience |
| “ There was a madam there who was telling me, instead of telling me…. You see my Strength had been finished, I mean completely finished, that they expected the baby was going to die so they pinched me even now I feel it being painful.” | You see my strength had been finished; they expected the baby was going to die so they pinched me; even now i feel it being painful | Physical abuse | Mistreatment during delivery | Neglect during delivery |
| “They should bring in equipment and materials necessary for Cesarean Section since they didn't have any. It can help in special cases.”  “: I remember I was bleeding so much I called the doctor and at that moment he attended to me immediately” | Should bring in equipment and materials necessary for Cesarean Section  I remember i was bleeding so much; I called the doctor; he attended to me immediately | Inadequate medical supplies  I called the doctor; he attended to me immediately | Facility culture/ environment  Responsive health workers | Negative experience  Positive experience |
| “he took the cotton wool and wiped me well and removed the blood clots that was inside and told me had I kept quiet I would have felt more pain, later he told me to sit down being that I was now fine” | He took the cotton wool and wiped me well; removed the blood clots that was inside; later he told me to sit down being that I was now fine | He took the cotton wool and wiped me well and removed the blood clots that was inside | Dignified care | Positive experience |
| “I felt bad because they were shouting on you and you are in the same room they didn’t want you to ……..even pushing the baby they didn’t you had to struggle alone.” | I felt bad because they were shouting on you; even pushing the baby they didn’t you had to struggle alone | Verbal abuse  Neglect during delivery | Mistreatment during delivery | Negative experience |
| “The way the serve their patients very fast they talk to them well and assist them to give birth well and is clean too they clean every time, their food is good and their place to rest is good there is no congestion there is no noise, yes.” | The way they serve their patients is very fast; they talk to them well; they assist them to give birth well; they clean every time; their food is good; there is no congestion; no noise | Timely service; cleanliness; good food; no congestion  They talk to them well | Facility culture/ environment  Respectful communication | Positive experience |
| “Hhmmm…… they gave me medicine, when I gave birth I had stomach pain and while giving birth they assisted me well, I did not have much pain. I don’t know how to explain it……...*laughs*………..they assisted me well.” | They gave me medicine; while giving birth they assisted me well | Gave me medicine  Assisted during delivery | Dignified care  Supportive care | Positive experience |
| “Things that showed me it is clean, in the side of the toilets was clean, the bed was clean, let’s say generally, not bedsheets …everything even the floor was very clean. You could even sit.” | In the side of the toilets was clean; the bed was clean; everything even the floor was very clean | Clean facility | Facility culture/ environment | Positive experience |
