# Supplementary material for: Exploring women’s childbirth experiences and perceptions of delivery care in peri-urban settings in Nairobi, Kenya
Source: Reprod Health. 2021 Apr 19;18:83. doi: 10.1186/s12978-021-01129-4 (PMC8054117; doi:10.1186/s12978-021-01129-4)
Supplement: Supplementary file 3 — Additional file 3. Coding Framework. [file 12978_2021_1129_MOESM3_ESM.docx]

**Appendix III**

**CODING FRAMEWORK AND FREQUENCIES FOR PCMC INTERVIEWS**

**THEME: RESPONSIVE HEALTH WORKERS**

| **Respondent ID** | **Include aspects of health worker being attentive, helpful etc** | **Non- responsive care. Include aspects of health workers not being keen to attend to patients** | **Facility** | **Not Applicable** |
| --- | --- | --- | --- | --- |
| 1 | When I called on him, he came immediately and helped. |  | private |  |
| 2 | The nurses treated us well, because whenever you said you had a problem they were there to help. |  | public |  |
| 3 |  |  | private | N/A |
| 4 | They attended to me the entire time, checking me from Time to time. |  | private |  |
| 5 |  |  | public | N/A |
| 6 | I mean when I called them they could come |  | private |  |
| 7 | the doctors were attentive. |  | public |  |
| 8 |  |  | public | N/A |
| 9 | I remember them calling for more help because the baby came out feet first, they came in quite a number of them. They came very fast. |  | public |  |
| 10 |  |  | private | N/A |
| 11 |  |  | private | N/A |
| 12 |  |  | private | N/A |
| 13 |  |  | public |  |
| 14 | .. I called the doctor who rushed in to help me. |  | public |  |
| 15 |  |  | public | N/A |
| 16 | There was a bed, oxygen machine,a man and woman who were checking me all the time. |  | public |  |
| 17 | they were attending to me any time I needed them |  | public |  |
| 18 |  |  | mission | N/A |
| 19 |  |  | public | N/A |
| 20 | Just their service when I got in there, they explained to me what I needed to know, because I was worried. |  | mission |  |
| 21 | they were attending to me any time I needed them |  | public |  |
| 22 |  |  | private | N/A |
| 23 | When they heard me cry of pain they came those two doctors they held my hands and told me we walk faster… they said the baby was coming out very fast they served me.  every time the doctors checks his works they keep on coming back to check on you until they make sure you are ok |  | public |  |
| 24 | The doctor and the nurse were present to attend to me. |  | private |  |
| 25 |  |  | public | N/A |
| 26 |  |  | public | N/A |
| 27 | in case you need something they provide it in time. |  | private |  |
| 28 |  |  | private | N/A |
| 29 |  |  | private | N/A |
| 30 |  |  | private | N/A |
| 31 | They received me and quickly attended to me. |  | private |  |
| 32 |  |  | private | N/A |
| 33 |  |  | private | N/A |
| 34 | They assessed me and did a quick scan because during that labour pains, the baby was moving up and down. |  | private |  |
| 35 |  |  | mission | N/A |
| 36 |  |  | public | N/A |
| 37 | I remember I was bleeding so much I called the doctor and at that moment he attended to me immediately |  | public |  |
| 38 | when you send the health providers they respond positively to your needs. |  | public |  |
| 39 |  |  | private | N/A |
| 40 |  |  | private | N/A |
| 41 | they assist people very fat and they keep on checking on you every time. |  | mission |  |
| 42 |  |  | mission | N/A |
| 43 |  |  | mission | N/A |
| 44 |  |  | private | N/A |
| 45 |  |  | private | N/A |
| 46 |  |  | private | N/A |
| 47 |  |  | private | N/A |
| 48 |  |  | private | N/A |
| 49 | They attended to me immediately I got there | there were some big doctors who were not even bothered.  they just leave you there until they hear you say you feel like the baby is coming, that is when they come.  .. | public |  |
| 50 |  |  | public | N/A |
| 51 |  | No one they come after you have finished | public |  |
| 52 | immediately I arrived, they started serving me. |  | private |  |
| 53 |  |  | public | N/A |
| 54 |  |  | private | N/A |
| 55 |  |  | private | N/A |
| 56 |  |  | private | N/A |
| 57 | they respond quickly. |  | private |  |
| 58 | they serve you as fast as possible |  | mission |  |
| 59 |  |  | public | N/A |
| 60 |  |  | public | N/A |
| 61 | she come quickly and help me, |  | public |  |
| 62 |  | when you call them I would say you just wait | public |  |
| 63 | she came immediately to help. |  | mission |  |
| 64 |  | when you feel too much pain and call they do not come, | public |  |
| 65 |  |  | public | N/A |
| 66 |  |  | mission | N/A |
| 67 |  | after admission he was told to go back home. | mission |  |
| 68 |  |  | mission | N/A |
| 69 | the doctors are very active there is no way you can go through many problems |  | mission |  |
| 70 |  |  | mission | N/A |
| FREQUENCY | 26 **(37%)** | 5 (**7%**) |  | 39 **(56%)** |

Responsive health workers per facility Non responsive health workers per facility

Private- 7 **(10%)** Private- 0 **(0%)**

Public- 14 **(20%)** Public- 4 **(6%)**

Mission- 5 **(7%)**  Mission- 1**(1%)**

**THEME: FACILITY CULTURE/ ENVIRONMENT**

| **Respondent ID** | **Include aspects of clean facility, given food, privacy, adequate staff, availability of equipment,** | **Poor facility culture- Include aspects of dirty facility, sharing beds, no privacy, inadequate equipment, long waiting time** | **Facility** | **Not Applicable** |
| --- | --- | --- | --- | --- |
| 1 | The hospital is very clean |  | private |  |
| 2 |  | The way they people share beds is not good, they do not use curtains for privacy, and mosquitoes bite the children.  When there was water shortage, the toilets would get dirty, making it hard to use them. | public |  |
| 3 | Cleanliness, the bed and food was brought for me and I showered with hot water then I was given drinking water. |  | private |  |
| 4 | Yes, they supported me, even gave me food |  | private |  |
| 5 |  |  | public |  |
| 6 | It was very clean.  -The bed I was given was nicely spread, it had a mosquito net so I saw it was good. |  | private |  |
| 7 | The surrounding was good it was clean |  | public |  |
| 8 | It was clean | people who admit patients Should be added like example you find that a heavy woman is waiting for a very long time to be admitted yet she is on pain | public |  |
| 9 | The room was very clean. |  | public |  |
| 10 | it was clean. | Maybe they be a little bit fast because they were few and they were interchanging the same personnel between the general ward and labour room. | private |  |
| 11 | The room was ok, it was very neat | there even if it’s the porridge is not hot its cold.  They should have their equipment.  when you get into a certain room you are told to give some money and yet it is written free.  They should have that security like when someone is giving birth they should be in their own closed place. It should not be open. | private |  |
| 12 | of the toilets was clean, the bed was clean | mosquito nets should be added and beds to should be added | private |  |
| 13 | My privacy was catered for. |  | public |  |
| 14 |  | They should hire more people to maintain the cleanliness and hygiene of the hospital.  It was not so clean.  The bed sheets were not clean, there were some funny smell in the room and generally the room looked untidy. | public |  |
| 15 |  | We were sharing beds  They were not clean (toilets)  Doctors should be added | public |  |
| 16 |  |  | public | N/A |
| 17 | Food was always served in time, and it was good food | The only challenges I faced were, waiting there for long | public |  |
| 18 | The facility is equipped |  | mission |  |
| 19 | It was clean and well arranged  They were also quick |  | public |  |
| 20 | It was smart and clean. |  | mission |  |
| 21 |  | The only challenges I faced were, waiting there for long | public |  |
| 22 |  |  | private | N/A |
| 23 | It was clean, I can’t lie it was very clean.  they give you food according to the quantity you request |  | public |  |
| 24 |  |  | private | N/A |
| 25 | it was clean it and wasn’t congested |  | public |  |
| 26 | I was given a bed where I was to deliver which was clean and good | The wait was long because we were many | public |  |
| 27 |  | Doctor should be added and the beds should also be added | private |  |
| 28 | I saw it was good it was clean there is water and the bed has a mosquito net  When I gave birth they gave me tea and bread I took. |  | private |  |
| 29 | the hospital was clean. |  | private |  |
| 30 | Clean facility |  | private |  |
| 31 |  |  | private | N/A |
| 32 |  |  | private | N/A |
| 33 | it is clean and they serve nice food. |  | private |  |
| 34 | Generally it is clean  their hygiene is on point  It was clean, the floor, bathrooms and toilets. Has nice food. | There should be enough number of doctors. | private |  |
| 35 |  |  | mission | N/A |
| 36 | It was good, even the toilets were clean | They should bring in equipment and materials necessary for Ceasarian Section since they didn't have any. It can help in special cases. | public |  |
| 37 | the room was clean |  | public |  |
| 38 | they attend to you in a friendly way they don’t make you to stay there for long |  | public |  |
| 39 |  |  | private | N/A |
| 40 |  | they do not admit any one giving birth to the first baby  they put you in a queue they are not in a hurry they go take tea and leave you there they don’t care.  They should add more health workers because they are few sometimes you get they give students who open you and stitch you in the wrong way, you know students have not qualified. | private |  |
| 41 | It was clean.  is clean too they clean every time,  The way the serve their patients very fast their food is good and their place to rest is good there is no congestion there is no noise, yes. |  | mission |  |
| 42 | I saw cleanliness  The room was clean. |  | mission |  |
| 43 | Those beddings are clean  Ruben is clean  spread the beds a mosquito net is there  The food you are given on time |  | mission |  |
| 44 | I did not wait for Long to be served |  | private |  |
| 45 | It was ok it was clean.  Food was given in time and with a good diet, hot shower and a soap. |  | private |  |
| 46 |  | Experienced doctors should be added | private |  |
| 47 |  | Doctors should be added | private |  |
| 48 |  | you get your injection outside the hospital.  they told me that food was over and I had to wait until night then they gave me ugali and kales | private |  |
| 49 |  | Where we were taken we were so congested so I had to sleep on the floor.    the place was dirty  there was no privacy.  But water…the water is so cold it just feels like it is from the freezer  we were so many women and the doctors were only 3. | public |  |
| 50 |  |  | public |  |
| 51 |  | there is no privacy, you are many.  the services there are bad.  they don’t have supervision for their workers  they should add more workers | public |  |
| 52 | The hospital is even cleaner than my house. |  | private |  |
| 53 |  |  | public |  |
| 54 | generally it is clean.  Has nice food | in my case there were few beds | private |  |
| 55 |  | Medicines should be added and also doctors | private |  |
| 56 | It was clean.  their services are good,their food was good |  | private |  |
| 57 | It was very clean |  | private |  |
| 58 | Their hygiene was great. |  | mission |  |
| 59 | could make sure every patient gets food  they were clean | the beds are few,  the bed I was we were two  I see if the doctors are few they should add more | public |  |
| 60 |  | had to wait for too long before being attended  It was dirty, they even left corpses of the dead babies there  If you give them some cash, they treat you well, make sure you get a bed and stay with you throughout.  They attend to you well if you have bribed them..  we were overcrowded yet there is covid 19 | public |  |
| 61 | I like the services and cleanliness | think they should have more doctors, beds | public |  |
| 62 |  | we were sharing one bed five mothers  some women were sleeping on the floor  Mama Lucy is dirty  If there are enough nurses, but nurses are overwhelmed | public |  |
| 63 | The facility was clean |  | mission |  |
| 64 |  | Yes there was there those who were sharing beds. whoever is there could see you. There is no privacy……  even under the bed they do not clean  There I did not see bed sheets.  I slept on a dirty bed on  Things like cup spoon there they sell and they sell them very expensive may be you didn’t go with it and they sell very expensive and then you do not have….  they should assist you with pampers for the baby, so people completely suffer other tell you to assist them with cotton wool.  it is dirty, the water is cold | public |  |
| 65 | there is enough equipment  It was clean  they were covering with those curtains  it has enough doctors and nurses | Congestion of ladies.  The beds were less. one bed they even share three people including children | public |  |
| 66 | the facility is clean  after you have given birth you shower with hot water | they should have more beds and pain killers. | mission |  |
| 67 | That room was nice and clean |  | mission |  |
| 68 | The room was smart and clean |  | mission |  |
| 69 | it is clean |  | mission |  |
| 70 | It is clean |  | mission |  |
| FREQUENCY | 45 **(64%)** | 28 **(40%)** |  |  |

Facility environment (+ve) per facility Facility environment (-ve) per facility

Private- 18 **(26%)** Private- 11 **(16%)**

Public- 15 **(21%)** Public- 16 **(23%)**

Mission- 12 **(17%)**  Mission- 1**(1%)**

**THEME: RESPECTFUL COMMUNICATION**

| **Respondent ID** | **Include aspects of how health worker communicated to the women** | **Poor communication** | **Facility** | **Not Applicable** |
| --- | --- | --- | --- | --- |
| 1 |  |  | private | N/A |
| 2 |  |  | public | N/A |
| 3 |  |  | private | N/A |
| 4 | Yes, the doctor were nice they were talking nicely to me |  | private |  |
| 5 |  |  | public |  |
| 6 | these do not insult people  From their talking, they were talking very well |  | private |  |
| 7 | doctors were good, they were not rude |  | public |  |
| 8 | They were caring and polite |  | public |  |
| 9 | They attended to me well and they did not yell or shout at me like I’ve heard. | Some doctors there are very harsh | public |  |
| 10 |  |  | private | N/A |
| 11 |  | Even you don’t have the strength to push the baby if you had your own problems when you tell when they tell you to sought out yourself.  She was told come here I test you or you go round and go where you wanted to go or you go back to where you came from it is not a must we serve you. | private |  |
| 12 | I was pleased by those nurses, that could talk to you as if they knew you. |  | private |  |
| 13 |  |  | public | N/A |
| 14 |  |  | public | N/A |
| 15 |  |  | public | N/A |
| 16 |  |  | public | N/A |
| 17 |  | After giving birth, the nurses at the ward were very rude, whenever you asked a question, they would answer you rudely, and most times it wasn’t the answer you were looking for. | public |  |
| 18 |  |  | mission | N/A |
| 19 |  |  | public | N/A |
| 20 |  |  | mission |  |
| 21 |  | nurses at the ward were very rude | public |  |
| 22 |  | Some rude nurses | private |  |
| 23 |  |  | public | N/A |
| 24 |  |  | private | N/A |
| 25 |  |  | public | N/A |
| 26 |  |  | public | N/A |
| 27 | They are very understanding |  | private |  |
| 28 |  |  | private | N/A |
| 29 |  |  | private | N/A |
| 30 | they talk to me in a respectful manner and were very patient |  | private |  |
| 31 |  |  | private | N/A |
| 32 | If I asked them something they were willing to answer.  they didn't abuse me or shouted at me |  | private |  |
| 33 | They treated me and the baby nicely, that's what I would call supportive care. |  | private |  |
| 34 | the doctors and nurses treated me nice |  | private |  |
| 35 | They were talking in a calm manner and respectful to me.  They served me with dignity |  | mission |  |
| 36 |  |  | public | N/A |
| 37 | the doctor’s talk to you well on the position to be in for the delivery to go on well |  | public |  |
| 38 |  |  | public |  |
| 39 |  |  | private |  |
| 40 |  | So I saw the way they were quarrelling people I said to myself I will not go back there again I decided to come here  When you are in pain they were talking harshly telling you not to shout, .  Yes they didn’t want if they hear you they would come and quarrel. | private |  |
| 41 | they talk to them well |  | mission |  |
| 42 | they were talking to her with respect |  | mission |  |
| 43 |  |  | mission | N/A |
| 44 |  |  | private | N/A |
| 45 |  | It was disastrous, you were shouted at  They didn't help me much because if I said what I wanted, other one kept shutting me up that I was disturbing people. | private |  |
| 46 |  |  | private | N/A |
| 47 |  |  | private | N/A |
| 48 |  | the nurses were threatening to leave if I didn't stop disturbing them.  They are just harsh on people | private |  |
| 49 |  | If you try and ask them again where is it they want you to go they respond rudely.  like now you are in pain and someone comes shouting telling you who asked you to stay on the beds  when I told her I am in pain she started saying I use drugs. | public |  |
| 50 |  |  | public | N/A |
| 51 |  | they get too harsh | public |  |
| 52 |  |  | private | N/A |
| 53 |  |  | public | N/A |
| 54 |  |  | private | N/A |
| 55 |  |  | private | N/A |
| 56 |  |  | private | N/A |
| 57 |  |  | private | N/A |
| 58 |  |  | mission | N/A |
| 59 |  |  | public | N/A |
| 60 |  | They did yell at me. When I said I was feeling pain and needed a pain killer, the attendant asked if my husband had paid for the injection. | public |  |
| 61 |  |  | public | N/A |
| 62 |  | nurse was very hash  if you try to talk with the doctor you are told to shut your mouth  male nurse also told me if I make noise I won’t deliver so I opted to keep quite. | public |  |
| 63 | none of them used any foul language when they were talking to me. |  | mission |  |
| 64 |  | They were rude  I went to them and called them they said I’m disturbing them  she is the one who even quarrels you | public |  |
| 65 | those were talking nicely to people |  | public |  |
| 66 |  |  | mission | N/A |
| 67 | they don’t insult people |  | mission |  |
| 68 |  |  | mission | N/A |
| 69 | they handle you well with care there is no raising of voices while talking to you or insulting you, yes |  | mission |  |
| 70 | They do not quarrel people they explain to you everything |  | mission |  |
| FREQUENCY | 20 **(29%)** | 13 **(19%)** |  |  |

Respectful communication per facility Poor communication per facility

Private- 8 **(11%)** Private- 5**(7%)**

Public- 5 **(7%)** Public- 8 **(11%)**

Mission- 7 **(10%)**  Mission- 0**(0%)**

**THEME: DIGNIFIED CARE**

| **Respondent ID** | **Include aspects of health worker being kind, friendly, caring, polite, nice, humane, feeling safe** | **Non dignified care- Include aspects of abuse** | **Facility** | **Not Applicable** |
| --- | --- | --- | --- | --- |
| 1 | I felt safe because I was with the midwife and I trusted everything was okay |  | private |  |
| 2 |  |  | public | N/A |
| 3 |  |  | private | N/A |
| 4 | Yes because the doctors were nice to me | They were nice except on doctor who was not friendly. | private |  |
| 5 |  |  | public |  |
| 6 | Yes, they were taking good care of me. |  | private |  |
| 7 | They were patience with me, if I asked questions regarding child birth they always helped, they were not arrogant. |  | public |  |
| 8 | This time they were kind and friendly  They were caring and polite |  | public |  |
| 9 | They were very friendly. |  | public |  |
| 10 | In other hospitals, there are rumours that caregivers mistreat women, they pinch the women. And none of that happened to me.  I was happy with their services. It made me feel like a woman.  Like the reception was great |  | private |  |
| 11 |  | they were just harassing me.  they pinched me even now I feel it being painful.  There are other doctors there who do not have that human heart. | private |  |
| 12 | they stitched me they finished then told the cleaner to give me water I shower. |  | private |  |
| 13 | Being treated nicely, being taken good care of. |  | public |  |
| 14 |  |  | public | N/A |
| 15 | they were nice |  | public |  |
| 16 |  |  | public | N/A |
| 17 | The doctors who were coming to check on us were very nice and kind and attended to us well |  | public |  |
| 18 | There services were good because, before delivery I had abdominal pain they treated me well, they attended to me nicely.  the doctor attended to me, they don't discriminate.  They attended to me nice |  | mission |  |
| 19 | The nurses were caring |  | public |  |
| 20 | The medical personnel serves faster and are so caring. |  | mission |  |
| 21 | The doctors who were coming to check on us were very nice and kind |  | public |  |
| 22 |  | Mean doctors | private |  |
| 23 | you will be served like a small baby |  | public |  |
| 24 | They attended to me well. |  | private |  |
| 25 |  |  | public | N/A |
| 26 | They treated me well |  | public |  |
| 27 | They nicely treat people  They were kind I was calmly welcomed |  | private |  |
| 28 |  |  | private | N/A |
| 29 | they were kind.  Yes I think they are professionals, they know exactly what they are doing. |  | private |  |
| 30 | The nurses were so friendly and so caring |  | private |  |
| 31 | They attended to me nicely |  | private |  |
| 32 | they were kind to me  they treat people nicely. |  | private |  |
| 33 | . |  | private | N/A |
| 34 | they treat people nicely, they are humane  the doctors and nurses have passion for what they are doing |  | private |  |
| 35 | They were welcoming and friendly. |  | mission |  |
| 36 | They were serving me nicely. They would take my blood pressure. They would also monitor the baby's movement  Because they treated me well | It's only the cook who had a not so pleasing attitude.  She would even remove the bedsheet that you are using to launder but will not replace. | public |  |
| 37 | the good thing was how the doctors were attending t the patient and the way they talk to patients in friendly manner I just felt it was okay |  | public |  |
| 38 |  |  | public |  |
| 39 |  |  | private |  |
| 40 |  | I felt bad because they were shouting on you and you are in the same room | private |  |
| 41 | They were treating me well, doing check-ups well and check-ups for the baby too |  | mission |  |
| 42 | They served her well |  | mission |  |
| 43 | I was served very well  the doctors are humble and caring is ok. they serve you very well with humility. |  | mission |  |
| 44 | The nurses were caring and polite |  | private |  |
| 45 |  |  | private | N/A |
| 46 |  |  | private | N/A |
| 47 | so that she can also get the nice treatment I got, they Even when I didn't have enough cash they were still patient With me |  | private |  |
| 48 | was just treated well  The nurses are good, |  | private |  |
| 49 |  |  | public | N/A |
| 50 | They treated me well  they supported and attended to me well. |  | public |  |
| 51 |  | that person will tell you they don’t want you to touch them, | public |  |
| 52 | they just served me well. |  | private |  |
| 53 | the services were good not bad |  | public |  |
| 54 | they treat people nicely, they are humane  they were encouraging me |  | private |  |
| 55 | They treated me nicely  the people there are caring, loving and conserned |  | private |  |
| 56 |  |  | private |  |
| 57 | The health worker respectable  they treated me well  They were nice. |  | private |  |
| 58 | They were serving me nicely and wholeheartedly.  doctors are good hearted |  | mission |  |
| 59 | the workers there are caring  they served me very well. |  | public |  |
| 60 |  | they did not attend to me well  was treated badly  . | public |  |
| 61 | I was treated well |  | public |  |
| 62 |  | when I tried to touch her she told me don’t touch me you have corona.  doctors don’t take care or even ask you how you are feeling they just pass you they tell you don’t touch me, | public |  |
| 63 | the nurses took very good care of me.  I was treated very well  the doctors and nurses there treat mothers with dignity, they are gentle and treat you with respect. |  | mission |  |
| 64 |  | They were not serving people in a good way  No you may even find they are seated talking and you go there they chase you away not that they are busy, | public |  |
| 65 | Their services are good they take their time to listen to the patient’s problems. |  | public |  |
| 66 | they handle women with care.  the doctors are caring. |  | mission |  |
| 67 | nurses and doctors are welcoming, loving and talk to people with respect.  that nurses talk to mothers with respect and they are humble.  I was treated with care |  | mission |  |
| 68 | I was really treated very well and with care,  They didn’t harass me the way I was harassed in the hospital where I delivered my first born. |  | mission |  |
| 69 |  |  | mission | N/A |
| 70 |  |  | mission | N/A |
| FREQUENCY | 47 **(67%)** | 9 **(13%)** |  |  |

Dignified care per facility Non-dignified care per facility

Private- 19 **(27%)** Private- 4 **(6%)**

Public- 17 **(24%)** Public- 5 **(7%)**

Mission- 11 **(16%)**  Mission- 0**(0%)**

**THEME: SUPPORTIVE CARE**

| **Respondent ID** | **Include aspects of birth companion, provision of birthing items, given pain medication, post delivery services** | **Lack of supportive care** | **Facility** | **Not Applicable** |
| --- | --- | --- | --- | --- |
| 1 | The nurse gave me some injection and instructed me to contact him when I noticed any changes. | No. other people were not allowed to be with me in the room. | private |  |
| 2 | I felt some pain. When I told the nurse, she gave me painkillers and an injection to help. | only the nurse was there. Family members were not allowed to be in there. | public |  |
| 3 | Yes, they gave me good services…… they gave me the medicine that is recommended, yes.  I was with a doctor and my parent. (during labor) |  | private |  |
| 4 | Yes, they supported me,even gave me food,Pampers, clothes, bathing water they were just nice. |  | private |  |
| 5 |  |  | public |  |
| 6 | Yes they took the baby, they put him on light, he was cleaned, dressed him up and I was taken to the bed.  they gave me blood boosting drugs, they told me how I should eat vegetables, how I will eat four times a day, they told me many things |  | private |  |
| 7 | Yes, I was supported by one staff to bed when I had serious labour pain, they even cleaned the place I delivered at. |  | public |  |
| 8 | They were taking good care of the babies while they were in there Nursery, I was also been given warm water for bathing and encouragements |  | public |  |
| 9 | They treated me well, after giving birth they even brought me food.  They really supported me. If it wasn’t for them, I wouldn’t have given birth well | They didn’t allow my mother to get into the delivery room. | public |  |
| 10 | There was a time the pain was too much that I couldn't get on top of the bed by myself and the nurse assisted me. |  | private |  |
| 11 |  | Not allowed to have a support person | private |  |
| 12 | Yes, when I was giving birth there was a nurse there, she was telling me to push, she was telling me when I feel the labor I’m supposed to push. So we assisted each other there and when it was going wrong she could tell me no do not do that |  | private |  |
| 13 | When I delivered, they helped me carry the baby and clothed it for me. |  | public |  |
| 14 |  | No person allowed | public |  |
| 15 |  | No person allowed | public |  |
| 16 |  | No person allowed | public |  |
| 17 |  | I did not feel supported, because they let me wait for too long and only helped me in the last minutes | public |  |
| 18 |  | No person allowed | mission |  |
| 19 |  | No person allowed | public |  |
| 20 |  | No person allowed | mission |  |
| 21 |  | I did not feel supported, because they let me wait for too long | public |  |
| 22 |  | No person allowed | private |  |
| 23 | when you are leaving they will not allow you to go alone with the baby they must make sure you have someone to take you up to your house. They can’t also allow you to go if they know you have a certain problem they will tell you to wait so that they can monitor you first  Yes they assisted me …told what to do |  | public |  |
| 24 |  | No person allowed- did not want | private |  |
| 25 | Was with the mother during labor  The doctor cleaned me after birth |  | public |  |
| 26 |  | I came with some family members, but they were not allowed to enter the room- did not want | public |  |
| 27 |  | No they were not allowed inside the room- did not want | private |  |
| 28 |  | No I was with the doctor alone...did not want | private |  |
| 29 | it was good since the nurse were always around asking how I feel and how the baby is doing.  They offered food, bedding and ensured that were I was sleeping was clean. |  | private |  |
| 30 | I was grateful because having your mum during delivery is a blessing.  I felt supported,each and every step I was supported because and they told me what to do. |  | private |  |
| 31 | they gave me folic acid and pain killers,they asked me to always go to the hospital in case I had any problem until I delivered, during delivery, they served me well | I was accompanied by family member, but they were not allowed in the labour room,they were told to wait outside. | private |  |
| 32 | I felt good, the doctors were free and my neighbour was not chased away even my husband was just at the door and was not chased away |  | private |  |
| 33 |  | They were not allowed in there. | private |  |
| 34 | When my legs grew numb, they were encouraging me to continue pushing! |  | private |  |
| 35 |  | No person allowed- wanted someone | mission |  |
| 36 | I came with someone who had been there before. She was the one who was looking for doctors. |  | public |  |
| 37 |  | No I never wanted anyone to be with me apart from the doctors | public |  |
| 38 | if you don’t know anything you are told maybe how to position your legs | the family member or friends are never allowed to in the room where one is delivering | public |  |
| 39 | Yes I was with my husband |  | private |  |
| 40 | I was served well after getting a baby, you are given hot water to shower and soap if you do not have. | No, they were told to go, there is another lady I found there who had been brought by another lady a friend of hers I presume but she was told to go away. They wanted you to stay alone but when they didn’t listen to what they were being told they were told to go somewhere else | private |  |
| 41 | They gave me medicine, when I gave birth I had stomach pain and while giving birth they assisted me well, I did not have much pain.  Washing a lady too, you see the way they wash someone inside there they do it slowly not painfully. |  | mission |  |
| 42 | Yes after giving birth is when I was injected | No, there was no one there only doctors.  you know it is a shameful thing you will not want him to see he will come to tell you the way you were doing, I wouldn’t want. | mission |  |
| 43 | they cleaned me they took me after finishing they took me into a shower room. I showered and after showering they gave me another cotton wool | if he is there he should not be close to where I am they put him a side shortly and wait. | mission |  |
| 44 | They helped me out with my baby and even showed me how to breast feed the baby | I would not have liked them to see me | private |  |
| 45 |  | : he was asked to go home. | private |  |
| 46 |  | Yes but due to the hospital rules that could not happen but I would have | private |  |
| 47 |  | No one my relatives and friends were outside  I would not like them to see me at that time | private |  |
| 48 |  | No I was only with the 2 nurses.  No I won't like to be accompanied. | private |  |
| 49 |  | there was no medicine that I was given.  I would not want any relative with me.  I was just alone  we were not given any medicines | public |  |
| 50 |  | No one from my family was there. | public |  |
| 51 |  | There they can’t allow anyone | public |  |
| 52 | I was with my husband |  | private |  |
| 53 | Yes I was with my husband |  | public |  |
| 54 |  | No, he was outside that room  I would not be comfortable ,and it is also not right | private |  |
| 55 |  |  | private |  |
| 56 |  | He was not allowed to stay  I would not like to be accompanied | private |  |
| 57 | gave me the right injections. | she remained outside the delivery room.  So I asked my sister to wait outside. | private |  |
| 58 | , they added some medicine to the drip  if you don't have clothes for the baby, they give you theirs which you retain even after being discharged.  . They never left but i stead encouraged me. | They were not present.  The administration also did not allow anyone else to be there. | mission |  |
| 59 | even give you pain killers | They said no with corona it will be difficult | public |  |
| 60 |  | Nobody gave me pain killers or even drip.  No, I was just alone  nobody was attending to me.  I was not supported | public |  |
| 61 |  | No I was alone my sister who took me to hospital didn’t enter the labor ward | public |  |
| 62 | I got support like when the doctor told me not to push I will tear, | No,no,no I was alone my mother in law was told to go back home from the gate  the nurses are not attentive  I had never seen the medicine which women are given to boost blood I was told to go and buy | public |  |
| 63 | The nurse was there encouraging me to push.  I was supported by the nurse.She then cleaned and dressed the baby after delivery. | nobody was allowed into the labour room. | mission |  |
| 64 |  | No, I wouldn’t have wanted. | public |  |
| 65 | they took the baby and washed him well and also the way I ….they stitched me they did not leave me like that they washed me I was bleeding a lot they injected me and gave me some medicine for pain and advised me what to do | No, they were not letting people in  Labor they do not give any medicine to reduce the pain | public |  |
| 66 | they are with you all the time while you are in labor pains | There was nobody it was me and the doctors alone. | mission |  |
| 67 | I bathed hot with hot water and then given hot tea.  I was given medicine to boost my blood level  they are welcoming |  | mission |  |
| 68 | they even washed for me my clothes ,cleaned my shoes, gave me some tea  the doctor even gave me some medicine to help boost my blood. |  | mission |  |
| 69 | The services there even after I finished giving birth they took the baby well and dressed him well after that you know also in the ward the way they take care of you they check on you they teach you how to breastfeed everything how |  | mission |  |
| 70 | Yes they supported me you see for the baby when you come out of theatre you are not able to cloth that is the first thing I found that they had clothed him very well and I was also clothed well so there is way they assisted me. |  | mission |  |
| FREQUENCY | 40 **(57%)** | 45 **(64%)** |  |  |

Supportive care per facility Non supportive care per facility

Private- 16 **(23%)** Private- 17 **(24%)**

Public- 13 **(19%)** Public- 20 **(29%)**

Mission- 10 **(14%)**  Mission- 8 **(11%)**
